# Bystander and abscopal effects of boron neutron capture therapy: radiobiological mechanisms and immunotherapeutic strategies

**DOI:** 10.3389/fonc.2026.1809510

**Published:** 2026-06-30

**Authors:** Zizhu Zhang, Chunyuan Zhao, Yizheng Chong, Longmin Li, Xiayang Zhu, Yujun Shao

**Affiliations:** 1 Department of Research and Education, Beijing Nuclear Industry Hospital, Beijing, China; 2 Nuclear Medicine Center, Beijing Nuclear Industry Hospital, Beijing, China; 3 Innovation Business Center, China Zhongyuan Engineering Corporation, Beijing, China; 4 Department of Cardiology, Beijing Nuclear Industry Hospital, Beijing, China

**Keywords:** abscopal effect, boron neutron capture therapy, bystander effect, high-LET radiation, radiation immunology

## Abstract

Boron neutron capture therapy (BNCT) is a high–linear energy transfer radiotherapy that enables cell-scale selective tumor killing and has shown clinical potential in recurrent and radioresistant cancers. While most BNCT research has focused on boron delivery, dosimetry, and direct local cytotoxicity, accumulating evidence suggests that BNCT can also elicit non-targeted biological responses beyond the irradiated volume. In this review, we summarize experimental and emerging clinical evidence indicating that localized BNCT induces both bystander and abscopal effects, manifested as biological alterations in neighboring non-irradiated cells and growth suppression of distant tumors. We further integrate these phenomena within a radiation immunology framework, proposing that high–linear energy transfer (high-LET)–induced immunogenic cell death, accompanied by the release of tumor antigens and damage-associated molecular patterns (DAMPs), reshapes the tumor immune microenvironment and drives systemic antitumor immune responses that mechanistically link bystander and abscopal effects. On this basis, we discuss the rationale for combining BNCT with immunotherapeutic approaches, positioning BNCT not only as a precision local radiotherapy but also as a potential trigger of systemic radio-immunological effects. This integrated perspective provides a conceptual framework for future mechanistic studies and the rational design of BNCT-based combination strategies.

## 1 Introduction

Boron neutron capture therapy (BNCT) is a biologically distinctive form of high–linear energy transfer (high-LET) radiotherapy that achieves selective tumor cell killing through the nuclear reaction between boron-10 (^10^B) and thermal or epithermal neutrons, generating high-energy α particles and ^7^Li nuclei with a path length comparable to the diameter of a single cell (1, 2). This unique microdosimetric feature allows BNCT to deliver lethal energy selectively to boron-enriched tumor cells while largely sparing surrounding normal tissues (3, 4). Over the past decades, advances in boron carrier development, neutron source technology, and treatment planning systems have facilitated the clinical translation of BNCT from reactor-based platforms to accelerator-based facilities, with encouraging results reported in recurrent head and neck cancer, malignant glioma, melanoma, and other radioresistant tumors (5, 6). Despite these advances, most BNCT-related studies have continued to emphasize boron pharmacokinetics, dosimetry optimization, and direct local cytotoxic effects, whereas the broader biological consequences beyond the irradiated volume remain incompletely understood (7, 8).

Historically, the earliest clinical experience with BNCT can be traced back to 1951, when neurosurgeon William Herbert Sweet and physicist Gordon Lee Brownell conducted the first clinical trial involving BNCT using the Brookhaven graphite research reactor (9–11). In 1968, Hiroshi Hatanaka initiated a clinical project in Japan. A retrospective review of the Japanese experience reported that, since 1968, 183 patients with various brain tumors were treated, and long-term survivors were identified (12, 13). Globally, approximately 26 accelerator-based BNCT (AB-BNCT) projects have been reported, including 15 focused on clinical applications and 11 dedicated to research purposes (14). Regarding treated tumor entities, clinical trials have primarily evaluated glioblastoma, head and neck carcinoma, meningioma, malignant melanoma, and liver cancer, with the scope expanding to lung cancer, breast cancer, extramammary Paget’s disease, osteosarcoma, clear cell sarcoma, and others (15). In Japan, where BNCT was approved for clinical use in 2020 for recurrent or locally advanced head and neck cancer, post-marketing surveillance involving 155 head and neck cancer cases reported an objective response rate of 72.3%, and a complete response rate of 46.0% (16). In China, BNCT has been explored in clinical studies involving recurrent head and neck malignancies, breast cancer, glioblastoma, and melanoma (17–19); however, most available evidence remains preliminary and should be interpreted cautiously.

It is now well established that bystander and abscopal effects are not unique to radiation therapy but represent general biological phenomena observed across diverse cancer treatment modalities, including chemotherapy, targeted therapy, and immunotherapy. In each case, treatment-induced stress in primary tumor cells can generate signals, such as soluble factors, extracellular vesicles, or immune cues, that alter the behavior of untreated neighboring or distant cells, and in some instances trigger systemic antitumor immunity. Parallel to advances in BNCT, radiation biology has increasingly recognized that the effects of ionizing radiation are not confined to directly irradiated cells. The concepts of the radiation-induced bystander effect and the abscopal effect have fundamentally challenged the traditional paradigm that equates radiation dose with localized biological outcome (20). The bystander effect refers to biological responses, such as DNA damage, chromosomal aberrations, and altered cell survival, occurring in non-irradiated cells that receive signals from irradiated neighbors, whereas the abscopal effect describes tumor regression at sites distant from the irradiated field (21). However, in the context of BNCT, these phenomena have often been mentioned only sporadically and lack a coherent conceptual framework.

Emerging experimental evidence suggests that BNCT is capable of inducing robust bystander and abscopal effects with potential therapeutic relevance. *In vitro* studies using co-culture and conditioned medium models have demonstrated that BNCT can provoke DNA damage, mutagenesis, and chromosomal instability in non-boron-loaded or non-irradiated neighboring cells, implicating gap junction intercellular communication, reactive oxygen species, soluble cytokines, and extracellular vesicles as mediators of bystander signaling (22, 23). *In vivo*, localized BNCT has been shown to suppress the growth of contralateral tumors and reduce metastatic burden in syngeneic tumor models, effects that are substantially diminished in immunodeficient animals, highlighting a critical role for the immune system. Moreover, preliminary clinical observations from BNCT combined with immunomodulatory approaches have reported regression or durable control of non-irradiated lesions in selected patients, although such evidence remains limited and largely anecdotal. Collectively, these findings imply that BNCT exerts biological effects that extend beyond direct tumor cell killing and may engage systemic antitumor mechanisms (24, 25).

From a radiation immunology perspective, the unique high-LET damage induced by BNCT provides a plausible mechanistic basis for integrating bystander and abscopal effects. High-LET radiation is known to efficiently induce immunogenic cell death, characterized by the release of tumor antigens and damage-associated molecular patterns (DAMPs), which promote dendritic cell maturation, T-cell priming, and reshaping of the tumor immune microenvironment. In this context, bystander signaling may represent local and regional amplification of stress and immune signals, whereas abscopal effects may reflect the downstream manifestation of systemic immune activation and immunological memory. This immunologically driven framework also helps to explain key differences between BNCT and conventional photon radiotherapy in terms of immune modulation and systemic efficacy, positioning BNCT as a potential initiator rather than a passive partner in radio-immunotherapy (26, 27).

In this review, we systematically summarize current evidence for BNCT-induced bystander and abscopal effects from *in vitro* experiments, animal studies, and early clinical observations, and integrate these findings within a unified radiation immunology framework. Furthermore, we discuss the biological rationale and emerging strategies for combining BNCT with immunotherapeutic approaches, including immune adjuvants and immune checkpoint inhibitors, with particular emphasis on treatment sequencing, patient selection, and appropriate biological endpoints. By highlighting non-targeted and systemic effects as central components of BNCT biology, this article aims to provide a conceptual basis for future mechanistic investigations and the rational design of BNCT-based combination therapies.

## 2 Radiobiological basis of BNCT and overview of systemic effects

### 2.1 Mixed radiation field and high-LET radiobiological characteristics of BNCT

From a radiobiological perspective, BNCT is fundamentally distinct from conventional photon radiotherapy due to the presence of a complex mixed radiation field. The therapeutic core of BNCT relies on the neutron capture reaction of boron-10 (^10^B), which generates high-LET α particles and recoiling ^7^Li nuclei (3, 28). These particles deposit their energy within an extremely short track length, comparable to the diameter of a single cell, resulting in highly localized but dense energy deposition predominantly confined to boron-enriched cells. In addition to the boron capture reaction, BNCT irradiation is accompanied by secondary components, including fast neutrons, γ rays, and reactions involving nitrogen, collectively contributing to the overall radiation field (29, 30).

This mixed-field composition underlies the unique biological effects of BNCT. Compared with low-LET radiation, high-LET particles induce complex and clustered DNA double-strand breaks, severe chromosomal aberrations, and molecular damage that is difficult to repair, thereby reducing the capacity for sublethal damage repair and increasing the probability of irreversible cell death. These characteristics explain the observed efficacy of BNCT in radioresistant and hypoxic tumors. A key distinction from low-LET radiation lies in the pattern of DNA damage: while low-LET photons generate predominantly isolated, reparable DSBs, BNCT-induced high-LET particles produce clustered lesions that are prone to misrepair and chromosomal aberrations, including micronuclei formation. This difference has direct immunological implications, as micronuclei are potent activators of the cytosolic DNA sensor cGAS-STING pathway, which bridges DNA damage to innate immunity. Importantly, however, the intense molecular damage initiated by BNCT is not restricted to directly irradiated cells, and its biological consequences may propagate beyond the primary target through secondary signaling mechanisms (1, 30).

### 2.2 Extension from local direct effects to non-targeted and systemic responses

Classical radiobiology has traditionally emphasized a direct relationship between physical dose and localized biological effects. This paradigm has been progressively revised with the recognition of non-targeted radiation effects, including the bystander effect and the abscopal effect, which demonstrate that radiation-induced responses can occur in cells and tissues not directly exposed to radiation (31). In the context of BNCT, the pronounced stress responses induced by high-LET damage provide a favorable biological environment for the amplification and dissemination of such effects, as demonstrated by the robust bystander responses observed after BNCT irradiation (32, 33).

Spatially, BNCT-associated biological responses can be conceptualized as a continuum. The initial level consists of direct high-LET cytotoxicity within boron-loaded tumor cells. This is followed by bystander responses in neighboring, non-irradiated cells, mediated by intercellular communication and soluble factors, as shown in BNCT co-culture models (32, 33). At a further level, these localized and regional signals may evolve into systemic responses, manifested as abscopal effects in distant tumors or metastatic sites (34). Temporally, BNCT initiates a cascade of immunomodulatory events. This process begins with the acute induction of stress responses and immunogenic signals within the tumor, which in turn can promote the recruitment and activation of immune cells in the local microenvironment. These changes suggest the potential to engage broader anti-tumor immunity (35, 36).

### 2.3 Potential biological and clinical significance of BNCT-induced systemic effects

The emergence of systemic effects has important implications for the therapeutic potential of BNCT. Within tumors, boron uptake and radiosensitivity are inherently heterogeneous, and bystander effects may partially compensate for insufficient boron accumulation or incomplete neutron exposure in certain tumor regions, thereby expanding the effective biological treatment volume (32, 33). Furthermore, in the setting of multifocal or metastatic disease, abscopal effects elicited by localized BNCT may contribute to the suppression of distant tumor growth, offering benefits that extend beyond the irradiated field, as shown in animal models.

At the same time, BNCT-induced systemic effects may also entail potential risks. Bystander signaling could induce DNA damage, genomic instability, or chronic inflammatory responses in adjacent normal tissues, raising concerns regarding late toxicity, a phenomenon observed in radiation-induced bystander effects (37). Similarly, excessive or dysregulated systemic immune activation may increase the likelihood of inflammatory or immune-related adverse effects, as noted in combination therapies involving radiation and immunotherapy (38). Therefore, a comprehensive understanding of the biological determinants of BNCT-induced systemic effects is essential for balancing therapeutic efficacy and safety.

### 2.4 Systemic effects of BNCT as a foundation for mechanistic and combination strategies

**Figure 1:**
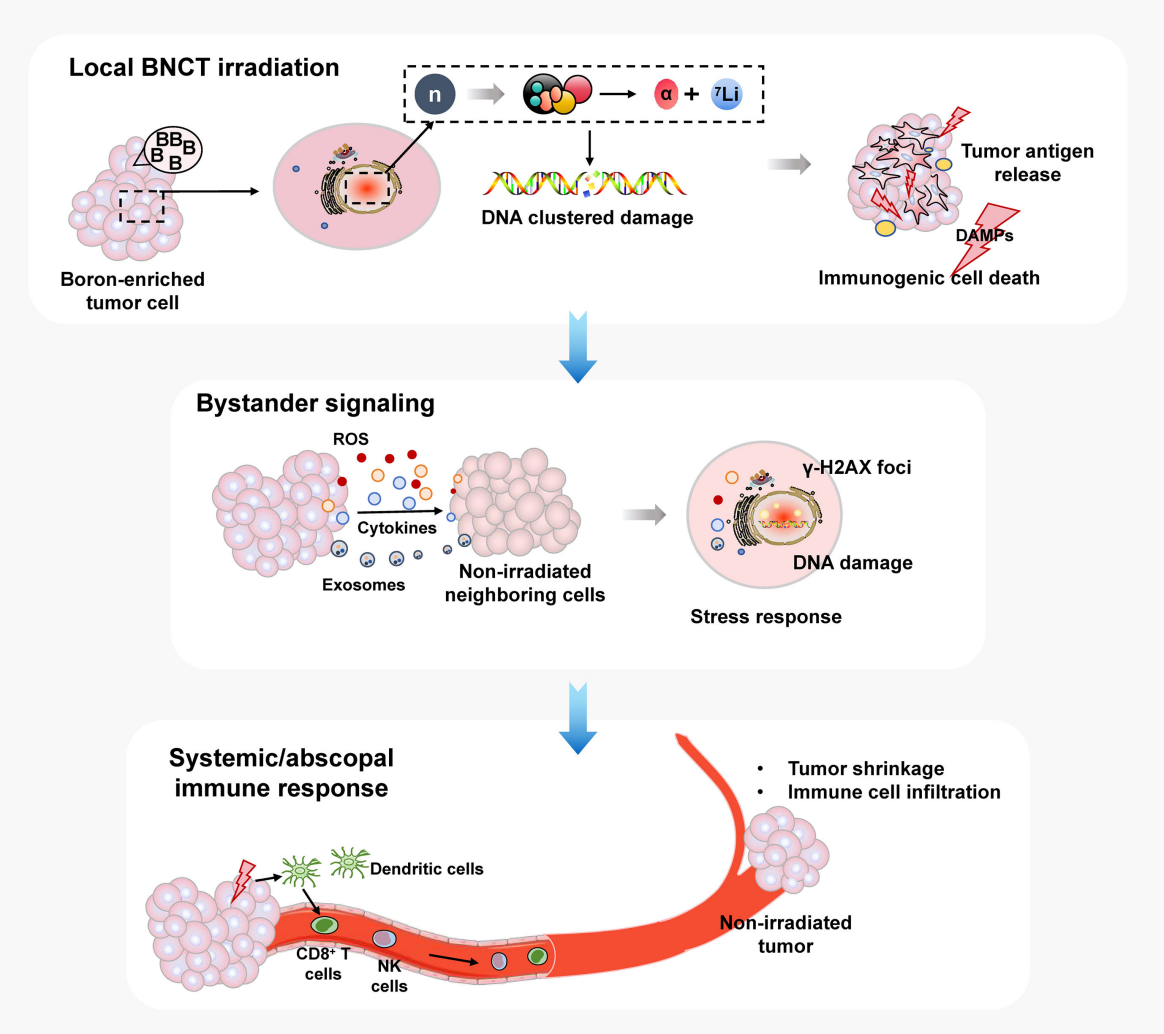
Schematic diagram of the systemic effect axis of BNCT local irradiation - bystander effect - distal effect.

Integrating bystander and abscopal effects into a unified framework of BNCT-induced systemic responses provides a conceptual foundation for subsequent mechanistic analyses and therapeutic strategies. Rather than being determined solely by physical dose distribution, the overall efficacy of BNCT may depend on the interplay between high-LET-induced primary damage, secondary signaling amplification, and immune-mediated regulation. This perspective naturally bridges BNCT radiobiology with radiation immunology and sets the stage for exploring immune-driven mechanisms underlying non-targeted effects, as demonstrated in BNCT studies combining immunomodulatory approaches (25, 39) (**Figure 1**).

Within this framework, BNCT emerges not merely as a localized precision radiotherapy, but as a potential initiator of systemic biological responses. Recognizing and characterizing this transition from local to systemic effects is a critical prerequisite for understanding BNCT-induced bystander and abscopal phenomena and for the rational development of BNCT-based immunotherapeutic combination strategies, which will be discussed in subsequent sections.

## 3 BNCT-induced bystander effects: experimental evidence

### 3.1 *In vitro* evidence for BNCT-induced bystander effects

Radiation-induced bystander effects are a well-recognized form of non-targeted biological responses and have been extensively investigated in both low- and high-LET radiation settings. In the context of BNCT, evidence for bystander effects has predominantly emerged from *in vitro* experimental systems designed to distinguish direct boron-dependent neutron capture damage from indirect signaling-mediated cellular responses.

Co-culture models, in which boron-loaded and non-boron-loaded cells are maintained in close physical proximity or share a common culture medium, have consistently demonstrated that BNCT induces biological alterations in cells not directly exposed to neutron irradiation or not containing boron (32). Under these conditions, only boron-enriched cells undergo direct high-LET damage, whereas neighboring cells function as bystanders.

Across multiple studies, BNCT-treated cultures have shown increased frequencies of hypoxanthine–guanine phosphoribosyltransferase (HPRT) mutations, elevated micronucleus formation, and enhanced γ-H2AX foci in bystander cells, indicating the induction of genomic instability and DNA damage responses beyond the directly targeted cell population (32, 33). Importantly, similar biological alterations have been observed when conditioned medium from BNCT-irradiated cells is transferred to non-irradiated recipient cells, even in the absence of direct cell–cell contact. These findings indicate that BNCT induces cytogenetic damage in non-irradiated cells, as assessed by mutation and micronucleus endpoints (32, 33, 40).

Collectively, these *in vitro* observations demonstrate that BNCT-induced high-LET damage initiates secondary signaling processes capable of propagating biological effects to non-targeted cells, thereby extending the impact of BNCT beyond regions of direct neutron capture.

### 3.2 Summary characteristics of BNCT-related bystander responses

Compared with bystander effects induced by conventional low-LET radiation, BNCT-associated bystander responses exhibit several distinctive features. First, the initiating damage originates from densely ionizing α-particles and recoiling ^7^Li nuclei generated by the ^10^B(n,α)^7^Li reaction, resulting in highly localized but intense molecular stress within boron-enriched cells (29, 41). Second, BNCT is characterized by a mixed radiation field, and the magnitude of bystander signaling may be influenced by boron distribution as well as contributions from accompanying neutron and γ-ray components.

Finally, BNCT-related bystander effects are frequently associated with persistent oxidative stress and genomic instability in recipient cells, suggesting that these responses may extend beyond transient signaling events and potentially contribute to long-term biological consequences (42, 43). These characteristics underscore the need to examine not only the existence of BNCT-induced bystander effects, but also the molecular mechanisms through which localized high-LET damage is translated into multicellular responses.

## 4 BNCT-induced bystander effects: possible mechanism pathways and biological implications

Building on the experimental evidence summarized above, this section focuses on the molecular and cellular pathways that mediate BNCT-induced bystander effects and their biological significance, rather than reiterating experimental endpoints. Current data indicate that BNCT-related bystander responses arise from the coordinated action of multiple intercellular communication mechanisms.

### 4.1 Gap junction intercellular communication

Gap junction intercellular communication (GJIC) represents a major contact-dependent pathway for the transmission of radiation-induced bystander signals. Gap junctions enable the direct transfer of small molecules, ions, and second messengers between adjacent cells, thereby facilitating rapid propagation of stress signals generated by localized high-LET damage (44).

Experimental inhibition of gap junctions has been shown to attenuate radiation-induced bystander endpoints in α-particle and other high-LET irradiation models (41, 43). Given that BNCT generates high-LET α-particles at the cellular level, similar mechanisms may plausibly contribute to BNCT-associated bystander responses, although direct experimental validation is still lacking.

### 4.2 Reactive oxygen species and redox-sensitive signaling

Reactive oxygen species (ROS) are central mediators of radiation-induced cellular stress and play a key role in bystander signaling across different radiation modalities (42, 43). Although direct evidence specifically in BNCT is limited, the high-LET α-particles generated by BNCT are expected to induce substantial ROS production, which may extend beyond the initial radiation track and contribute to bystander effects, as observed in α-particle irradiation studies (42).

Bystander cells exposed to conditioned medium from irradiated cultures exhibit increased oxidative stress markers and DNA damage, effects that can be partially mitigated by antioxidant treatment (43, 45). These observations indicate that ROS and redox-sensitive signaling pathways contribute to the initiation, amplification, and maintenance of radiation-induced bystander responses, and likely play a role in BNCT-induced bystander effects as well. Furthermore, micronuclei arising from BNCT-induced clustered damage in directly irradiated cells can, upon nuclear envelope rupture, release genomic DNA into the cytoplasm of the same cell, activating the cGAS-STING pathway and promoting the secretion of inflammatory cytokines such as IL-6, TNF-α, and type I IFNs. These soluble mediators then act as bystander signals that induce DNA damage and inflammatory responses in neighboring non-irradiated cells, providing a direct mechanistic link between high-LET-induced cytogenetic lesions and the propagation of bystander effects.

### 4.3 Soluble factors and extracellular vesicles

In addition to direct cell-cell communication and oxidative signaling, soluble mediators released from irradiated cells are increasingly recognized as important contributors to bystander effects (46–48). Cytokines and growth factors, including interleukin-6 and transforming growth factor-β, have been implicated in radiation-induced bystander signaling and may also participate in BNCT-related responses by modulating DNA damage repair, inflammatory pathways, and cell survival.

More recently, extracellular vesicles, particularly exosomes, have emerged as potential carriers of complex bystander signals (49, 50). These vesicles can encapsulate proteins, nucleic acids, and metabolites released from damaged cells and deliver them to non-irradiated recipient cells, thereby inducing phenotypic and molecular changes. Although direct evidence specifically linking extracellular vesicles to BNCT-induced bystander effects remains limited, their established role in radiation-induced intercellular communication supports a plausible and biologically relevant mechanism that warrants further investigation in the BNCT context.

### 4.4 Biological implications for tumor control and normal tissue response

The existence of BNCT-induced bystander effects has important biological and clinical implications. In tumor tissues, heterogeneous boron uptake and uneven neutron distribution represent major challenges for achieving uniform tumor control. Bystander signaling may partially compensate for these limitations by extending biological damage beyond regions of optimal boron accumulation, thereby increasing the effective treatment radius and potentially improving local tumor control probability, as suggested by studies on radiation-induced bystander effects (32, 33, 51).

Conversely, bystander effects in adjacent normal tissues raise theoretical concerns regarding delayed toxicity and genomic instability, as observed in radiation-induced bystander responses (37, 52). DNA damage and stress responses induced in non-irradiated normal cells may contribute to late radiation effects, particularly at tumor-normal tissue interfaces. These considerations highlight the potential need to account for bystander responses when defining target margins and protecting organs at risk in BNCT treatment planning, though direct evidence in BNCT is currently limited.

## 5 BNCT-induced abscopal effects and systemic antitumor responses

### 5.1 Evidence from animal models

Direct support for BNCT-induced abscopal effects has mainly come from well-controlled animal studies in which radiation exposure is intentionally confined to a single lesion while distant tumors or metastatic sites are left untreated. In bilateral tumor models, BNCT delivered to only one tumor consistently results in a measurable growth delay or suppression of the contralateral, non-irradiated tumor (39). A key biological feature emerging from these experiments is the dependence on host immune competence: abscopal suppression is markedly weakened or lost in immunodeficient animals, indicating that the distant antitumor effect is not a simple extension of local radiation injury but instead requires an intact immune system to translate local BNCT-induced damage into systemic tumor control (25, 39).

Metastatic models provide complementary evidence that strengthens the concept of a BNCT-driven systemic mechanism. Following localized BNCT to a primary tumor or a dominant lesion, studies have reported reductions in the number and/or volume of lung metastatic nodules, while pulmonary toxicity remains controllable within the tested regimens (53). These findings are biologically meaningful because metastatic deposits are outside the irradiated field and cannot be explained by direct physical dose. Instead, the observed suppression of distant metastases suggests that localized BNCT may influence the progression of disseminated disease in specific experimental setting, thereby positioning abscopal effects as a clinically relevant extension of BNCT biology rather than a rare experimental curiosity.

### 5.2 Immune and systemic changes associated with abscopal effects

Mechanistically, the abscopal effects observed after BNCT are consistently accompanied by systemic immune alterations that align with a model of immune-mediated tumor control. Across multiple preclinical settings, increases in peripheral CD8+ T-cell proportions and activation markers have been reported, together with enhanced natural killer (NK) cell activity, suggesting amplification of cytotoxic effector function at the systemic level after BNCT (54).

Beyond peripheral blood, immune remodeling in lymphoid and tumor-draining compartments further supports the systemic nature of BNCT-induced responses. Collectively, these systemic and immune readouts provide convergent evidence that BNCT-associated abscopal effects are tightly linked to systemic antitumor immunity, bridging local high-LET injury to distant tumor suppression through immune activation, trafficking, and effector execution (55, 56).

### 5.3 Clinical signals and current limitations

Clinical evidence for BNCT-related abscopal effects remains preliminary but provides important signals that justify further investigation, particularly in the context of combination therapy. Case-level observations have described regression, stabilization, or prolonged control of non-irradiated lesions in patients receiving BNCT, with reports in malignant melanoma and extramammary Paget’s disease (57, 58). However, these observations are largely anecdotal and do not specifically involve combination with immune checkpoint inhibitors. To systematically assess the available clinical evidence, **Table 1** summarizes the historical clinical observation results related to BNCT, while distinguishing between direct distant effect evidence, supportive local reaction evidence, and confounding factors of pseudo-distant effect. A critical consideration in interpreting the limited clinical evidence for BNCT-induced abscopal effects is the need to rigorously exclude “pseudo-abscopal” confounders—that is, non-immune-mediated distant responses that could be erroneously attributed to systemic immunity. Two sources of such confounding are particularly relevant to BNCT. First, unintended physical dose from neutron scattering, also referred to as stray radiation, may deposit low but measurable doses in tissues outside the intended irradiation field, especially when BNCT is delivered using reactor-based neutron sources. This scattered dose, while minimal, could contribute directly to cell killing in distant lesions and thus mimic an abscopal effect without involving immune mechanisms. Second, circulating boron-10 (^10^B) compounds in the bloodstream may be present throughout the body during neutron beam irradiation. Boron that is not specifically localized to the target lesion—whether remaining in the circulation or taken up by other tissues—can undergo neutron capture reactions elsewhere in the body, generating localized high-LET damage at non-targeted sites. This so-called “circulating boron effect” could produce direct radiological cell killing at distant locations, confounding the attribution of distant tumor regression to immunologically mediated systemic responses.

**Table 1:** Selected clinical observations potentially relevant to systemic or distant responses after BNCT.

Study	Country	Tumor type / patients	BNCT setting	Reported clinical response	Abscopal interpretation	Key pseudo-abscopal confounders/limitations
Hiratsuka et al., 2018 (57)	Japan	Vulvar melanoma and genital extramammary Paget’s disease; 4 patients	BPA-BNCT	All patients achieved complete response within 6 months	Clinical BNCT response evidence; not definitive abscopal evidence	Lesions appear to have been included in treatment field; no systematic distant-lesion endpoint; no immune monitoring
Hiratsuka et al., 2020 (58)	Japan	Cutaneous melanoma; 8 patients / 8 lesions	BPA-BNCT	ORR(CR + PR without recurrence): 88 % (8/7)	Strong local-control evidence, but not abscopal evidence	Patients had no nodal or distant metastases; primary treated lesions only; no non-irradiated lesion endpoint
Kashihara et al., 2025 (78)	Japan	Cutaneous angiosarcoma and malignant melanoma; 10 patients	BPA-BNCT (accelerator-based)	ORR: 70 % (median tumor shrinkage rate: 77.5 %)	Recent clinical safety/feasibility context	Local treatment study; no immune-mediated distant response endpoint
Menendez et al., 2009 (79)	Argentina	Stage IV melanoma with multiple metastases; 7 patients	BPA-BNCT	Lesion-based CR + PR: 69.3%	Relevant metastatic clinical context, but not definitive abscopal evidence	Multiple lesions/metastases; response assessed per treated lesion; possible scattered dose/circulating boron contribution not excluded; no systematic immune monitoring
Koivunoro et al., 2019 (80)	Finland	Locally recurrent head and neck squamous cell carcinoma; 79 patients	BPA-BNCT	CR: 25, PR: 22, SD: 17, PD: 5	No abscopal evidence; local response only	Single-arm, single-center; no distant lesion endpoint; no immune monitoring; scattered dose not excluded
			1–2 fractions BNCT			
Kato et al., 2009 (81)	Japan	Advanced recurrent head and neck cancer; 26 patients	BPA-BNCT	CR: 12, PR: 10, PD: 3; ORR 85%	No abscopal evidence; local response only	Small sample; no predefined out-of-field endpoint; circulating boron not excluded
Kageji et al., 2011 (82)	Japan	Glioblastoma multiforme; 23 patients	BSH +BPA BNCT (intraoperative or non-surgical)	survival time:19.5 months; 2-, 3-, and 5-year survival rates were 26.1%, 17.4% and 5.8%, respectively	No abscopal evidence; local CNS response only	Single institution; no distant lesion endpoint (CNS disease confined to brain); circulating boron possible but CNS penetration limited

ORR, overall response rate; CR, complete response; PR, partial response; SD, Stable disease; PD, Progressive disease; CNS, central nervous system.

To rigorously delineate true immune-mediated abscopal effects from these direct radiological confounders, future clinical studies should incorporate the following measures (1): detailed dosimetric characterization of neutron scattering to quantify unintended doses to distant organs (2); measurement of blood boron levels at the time of neutron irradiation to estimate the potential contribution of circulating boron-induced damage (3); comparison of distant lesion responses between immunocompetent and immunodeficient animal models under identical irradiation conditions; and (4) inclusion of immune monitoring (e.g., T-cell activation markers, cytokine profiling, tumor-infiltration lymphocyte analysis) to provide functional evidence of systemic immune engagement. In the absence of such controls, the handful of clinical anecdotal reports of distant responses after BNCT cannot be reliably distinguished from these pseudo-abscopal phenomena. This critical distinction is essential for advancing BNCT from a purely local modality to a truly systemic radio-immunotherapy strategy and for the rational design of BNCT-based combination regimens with immune checkpoint inhibitors.

These findings are consistent with an immune-mediated interpretation and align conceptually with preclinical data suggesting that BNCT can function as a trigger for systemic antitumor responses (25, 39). Nevertheless, current clinical support is limited by the small number of reported cases, heterogeneity in tumor types and treatment protocols, and the lack of standardized endpoints or immune monitoring specifically designed to capture abscopal biology. Consequently, the presence, frequency, and determinants of BNCT-induced abscopal effects cannot yet be reliably quantified. As summarized in **Table 1**, none of the historical clinical reports included systematic out-of-field dosimetry or immune monitoring to exclude pseudo-abscopal confounders. Therefore, prospective studies with predefined distant-lesion endpoints, systematic immune profiling, and rigorous documentation of treatment sequencing are therefore required to validate these early clinical signals and determine whether abscopal effects can be reproducibly leveraged in BNCT-based therapeutic strategies (38).

## 6 Immunomodulation and BNCT-based combination strategies with immunotherapy

### 6.1 Immunogenic cell death and remodeling of the tumor immune microenvironment

A key biological feature distinguishing boron neutron capture therapy (BNCT) from conventional low-LET radiotherapy lies in its ability to convert highly localized physical damage into immunologically meaningful signals. During BNCT, the 10B(n,α)7Li reaction generates densely ionizing α particles and recoiling 7Li nuclei, which produce complex clustered DNA damage that is difficult to repair. This damage pattern is qualitatively different from the isolated, more repairable double-strand breaks induced by low-LET photons (e.g., X-rays or γ-rays) (28, 59–63). This damage induces more complex chromosomal aberrations, increasing the possibility of chromosomal fragments not integrating into the main cell nucleus during mitosis, thereby promoting micronucleus formation (64, 65) and triggering subsequent immune recognition responses. At the DNA-sensing level, micronuclei derived from unrepaired chromosomal damage may undergo nuclear envelope rupture, allowing genomic DNA to accumulate in the cytoplasm. Cytosolic DNA is recognized by cyclic GMP-AMP synthase (cGAS), which catalyzes the production of 2′,3′-cGAMP and activates stimulator of interferon genes (STING). This process triggers downstream TBK1–IRF3 signaling and induces type I interferons and pro-inflammatory cytokines (65–71). Thus, the cGAS-STING pathway functions as a central molecular bridge linking BNCT-induced high-LET DNA damage to innate immune activation. Compared with low-LET radiation, the denser and more complex DNA lesions induced by BNCT-induced high-LET particles may provide a stronger biological basis for sustained cytosolic DNA sensing and immune stimulation (72). At the level of immunogenic cell death (ICD), BNCT-induced irreversible tumor cell injury may promote the release or surface exposure of damage-associated molecular patterns (DAMPs), including HMGB1, ATP, and calreticulin (54). These DAMPs serve as danger signals that facilitate dendritic cell recruitment, maturation, antigen uptake, and antigen presentation, thereby supporting the priming of tumor-specific T cells. In this process, BNCT is not merely a local cytotoxic modality but may also function as an immunological trigger that transforms tumor cell death into adaptive antitumor immune responses. Experimental evidence supports this mechanism framework. It has been reported that BNCT can promote the accumulation of cytoplasmic double-stranded DNA in colorectal cancer cells and the upregulation of IFNβ, as well as a manganese-based nano-boron agent being able to synergize with BNCT-induced oxidative stress to enhance cGAS-STING signaling, dendritic cell maturation, and the immune response of CD8^+^ T cells (59, 70). Additionally, in tumor models, immune remodeling phenomena related to BNCT have been observed, including enhanced inflammatory signals and enhanced anti-tumor immune responses, supporting the view that BNCT can reshape the tumor immune microenvironment to be more conducive to immune responses (39). These representative findings indicate that the DNA damage, innate immune sensing, and ICD caused by BNCT are interrelated rather than independent events.

Collectively, BNCT-induced immune remodeling can be understood as a stepwise process: first, high-LET radiation causes dense and complex DNA damage; second, unrepaired chromosomal fragments and micronuclei activate cytosolic DNA-sensing pathways; third, ICD-associated DAMPs and inflammatory cytokines promote dendritic cell activation and T-cell priming; and finally, local immune activation may propagate to neighboring non-irradiated cells and distant tumor sites. This layered mechanism provides a biological foundation for both bystander and abscopal effects and supports the rationale for combining BNCT with immunotherapeutic strategies.

### 6.2 Rationale for combination strategies from the perspective of bystander and abscopal effects

When viewed through the lens of bystander and abscopal biology, BNCT exhibits several features that make it particularly suitable for combination with immunotherapeutic approaches. At the local and regional level, bystander effects reflect the spread of stress signals and immune activation to neighboring, non-irradiated cells and tissues (32, 33). This phenomenon may partially compensate for heterogeneous boron distribution and variable radiosensitivity within tumors, effectively broadening the biological impact of BNCT beyond regions of optimal neutron capture.

At the systemic level, abscopal effects represent the culmination of immune-mediated tumor control at distant sites and are closely associated with the generation of systemic antitumor immunity and immunological memory (34). For patients with multifocal or metastatic disease, such systemic responses offer a potential therapeutic advantage that cannot be achieved through localized treatment alone. Importantly, the ability of BNCT to induce robust antigen release and danger signaling positions it as an effective ‘radio-immunological ignition point,’ capable of converting an immunologically cold tumor into one that is responsive to immune checkpoint inhibition or immune adjuvants, as suggested by preclinical models (39, 73, 74).

### 6.3 Preclinical and early clinical exploration of BNCT–immunotherapy combinations

Preclinical studies provide initial support for the synergistic potential of BNCT combined with immunomodulatory interventions. In animal models, BNCT in combination with bacillus Calmette-Guérin (BCG) or immune adjuvants has been shown to enhance suppression of both irradiated and contralateral tumors, improve overall survival, and strengthen systemic immune activation (25).

In selected patients treated with BNCT, favorable local control and indications of distant lesion stabilization or regression have been reported (58, 75). While these observations remain preliminary and heterogeneous, and do not specifically involve combination with modern immunotherapies such as PD-1/PD-L1 inhibitors, they are consistent with the immunological mechanisms inferred from preclinical models and support continued investigation of BNCT as a partner modality in combined radio-immunotherapy.

### 6.4 Conceptual framework for BNCT-based combination strategies guided by bystander and abscopal effects

Building on current evidence, several strategic considerations emerge for the rational design of BNCT-based combination therapies. With respect to treatment sequencing, a biologically intuitive approach is to deliver BNCT first to induce tumor antigen release and DAMP exposure, followed by concurrent or sequential administration of immune checkpoint inhibitors or other immunotherapies to amplify and sustain antitumor immune responses (38, 76). Such sequencing aligns with the temporal dynamics of ICD and immune priming observed in radiation-immunotherapy combinations.

**Figure 2:**
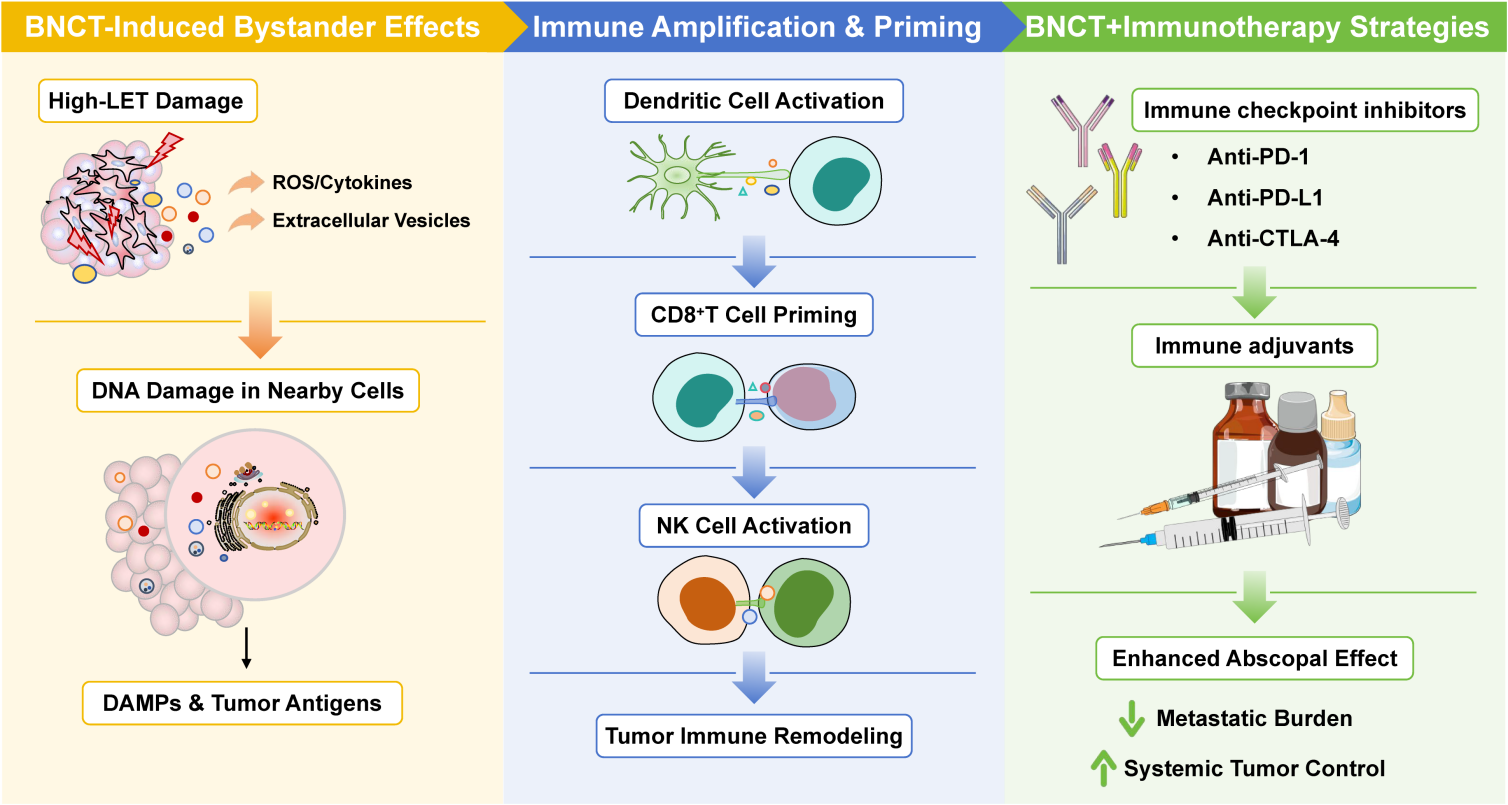
The framework of BNCT + immunotherapy strategy based on the bystander effect and the remote effect.

Patient and lesion selection also warrant careful consideration. Individuals with higher metastatic burden or tumors exhibiting baseline immune infiltration may be more likely to benefit from BNCT combined with immunotherapy, as these conditions provide a receptive immunological context for systemic response amplification (38, 77). Finally, future studies should incorporate endpoints that explicitly capture bystander- and abscopal-related outcomes, including responses in contralateral or distant lesions and longitudinal immune phenotyping, rather than relying solely on response rates at the primary irradiated site. Together, these considerations outline a framework in which bystander and abscopal effects are not incidental observations, but central design principles guiding the development of BNCT-based radio-immunotherapy strategies (**Figure 2**).

## 7 Conclusion

BNCT may extend beyond a purely local modality: it is not merely a high-LET radiotherapy based on physical precision, but also a treatment mode that can trigger systemic biological effects through non-targeted and immune-mediated responses. Experimental evidence shows that BNCT can extend the local biological effects of high-LET damage to non-irradiated areas through bystander effects and distant effects, and activate systemic anti-tumor immune responses. Although the clinical translation evidence of these effects is still accumulating, especially the potential for combination with immunotherapy remains to be fully verified, this perspective has provided a new framework for a comprehensive understanding of the mechanism of BNCT. In the future, standardized research is needed to quantitatively evaluate the intensity and safety of these systemic effects, and develop corresponding biomarkers, so as to promote BNCT from a local treatment method to a truly systemic radio-immunotherapy strategy.
